# Striped Non-Uniform Corrosion Behavior of Non-Equiatomic FeMnCoCr High-Entropy Alloy Prepared by Laser Melting Deposition in 0.1 M H_2_SO_4_ Solution

**DOI:** 10.3390/ma13235554

**Published:** 2020-12-05

**Authors:** Zhijian Zhang, Tiechui Yuan, Ruidi Li

**Affiliations:** 1 State Key Laboratory of Powder Metallurgy, Central South University, Changsha 410083, China; zhangzhijian@csu.edu.cn; 2 Key Laboratory of Functional Metal-Organic Compounds of Hunan Province, Hengyang Normal University, Hengyang 421001, China

**Keywords:** high-entropy alloy, laser melting deposition, polarization, EIS, corrosion behavior

## Abstract

The corrosion behavior of the Fe_50_Mn_30_Co_10_Cr_10_ high-entropy alloy (HEA) manufactured via laser melting deposition (LMD) was investigated using open circuit potential, potentiodynamic polarization, and electrochemical impedance spectroscopy measurements. The microstructure and morphology of LMD samples before and after the electrochemical tests were compared using X-ray diffraction, optical microscopy, scanning electron microscopy, and electron backscatter diffraction techniques. After the corrosion tests, a striped morphology was observed on the surface of the LMD HEA, which is mainly caused by the interval distribution of high-density and low-density grain-boundary regions. The corrosion performances varied with different planes of the LMD HEA sample, which is mainly controlled by the grain size at each plane. Local corrosion in this HEA is concentrated at the melt pool boundary, which may be due to the abundant metallurgical defects and stress concentration at this location.

## 1. Introduction

High-entropy alloys (HEAs) are alloys with multiple principal elements. Because of their high configurational entropy and low enthalpy of mixing [1], HEA can form not only a simple phase of FCC and BCC but also a simple phase of HCP [2,3]. A large number of studies have shown that these alloys generally have excellent mechanical properties and high thermal stability [1,4,5,6,7,8,9,10]. In recent years, a Fe_50_Mn_30_Co_10_Cr_10_ non-equiatomic dual-phase HEA was proposed, which has attracted the attention of materials scientists due to its high strength and good plasticity [11,12,13,14]. The excellent and comprehensive mechanical properties of this HEA are mainly due to the release of internal stress and the formation of ductile austenite.

Typically, HEAs are manufactured by casting and mechanical deformation [15], which are difficult to prepare components with ultrafine grains and/or complex shapes. However, additive manufacturing (AM) technology can be used to tackle the aforementioned issue. Laser melting deposition (LMD) is a typical powder-fed AM technology with a layer wise feature [16,17,18], and it shows advantages, such as raw material saving and near-net shaping with high density part. This technology has been utilized to process various metal powders in recent decades [19,20,21,22,23,24,25].

Corrosion is a key performance factor that limits the application range of many materials. However, the corrosion performance of this material, which has a wide application prospect, has not been studied. In recent years, a lot of research has been conducted on the corrosion properties of HEAs [1,25,26,27,28,29,30,31,32,33]. Most of them are single phase HEAs with near-equal proportions of elements. There is no obvious regularity in the corrosion performance of various HEAs, and their corrosion resistances are also very different.

HEAs usually have good corrosion resistance, which is mainly due to their high entropy. In general, the high entropy effect makes the alloy not easily able to produce composition segregation and precipitated phase, avoids the production of micro-couple, and reduces the corrosion-related electrochemical reactions. In addition, due to the great variability of the principal component of HEA, the composition of HEA can be easily adjusted. It is worth mentioning that the corrosion resistance of HEA can be further improved by adding corrosion resistant elements. The addition of common corrosion-resistant elements such as Cr, Ti, and Mo usually leads to the formation of a passive film and thus improves the corrosion resistance of the alloy.

To the best of our knowledge, there have been no reports regarding the preparation of this HEA by LMD or studies of its corrosion performance. In this study, we used LMD technology to prepare non-equiatomic dual-phase Fe_50_Mn_30_Co_10_Cr_10_ samples and investigated their corrosion properties at different planes in 0.1 M H_2_SO_4_ solution.

## 2. Materials and Methods

### 2.1. Material Preparation

**Figure 1:**
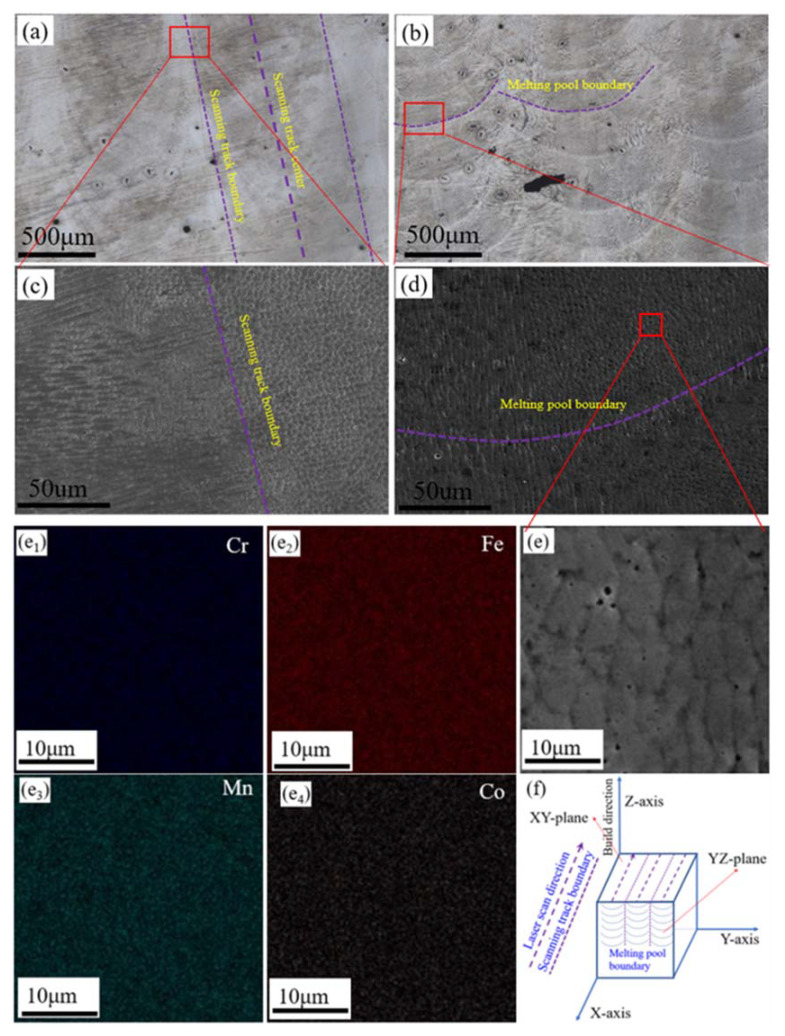
Microstructures and schematic diagram of the laser melting deposition (LMD)-produced Fe_50_Mn_30_Co_10_Cr_10_ HEA cube: (**a**,**b**) OM images of the XY- and YZ-planes; (**c**,**d**), SEM images of the XY- and YZ-planes; (**e**,**e_1_**−**e_4_**) SEM image and EDS mapping results of the corresponding area in (**d**); and (**f**) schematic diagram of the cube.

The non-equiatomic Fe_50_Mn_30_Co_10_Cr_10_ HEA powders were prepared by gas atomization in a high-purity argon atmosphere and powders with particle size between 75 μm and 150 μm were selected for LMD processing. The chemical composition of the raw powder was measured by inductively coupled plasma-atomic emission spectroscopy, as shown in Table 1. The LMD sample was fabricated using an RC-LDM8060 machine (Zhongkeyuchen, Nanjing, China) equipped with a 2000-W fiber laser with a spot size 2 mm in diameter. During the LMD process, the samples were produced in an N_2_ atmosphere with oxygen content less than 0.05%. We used 45# steel plate as the building substrate. During LMD, the scanning direction was maintained at a 0° angle rotation for two successive layers, as shown in Figure 1. The selected laser power, scan speed, hatch spacing, and layer thickness were 1000 W, 800 mm/s, 1 mm, and 0.3 mm, respectively. In this work, we used a LMD Fe_50_Mn_30_Co_10_Cr_10_ HEA cube with the dimensions 20 mm × 20 mm × 20 mm. Samples with the dimensions 10 mm × 10 mm × 1 mm were cut from the cubes for the XY- and YZ-planes. All samples were mechanically grounded to 2000 grit using SiC paper and then polished with a suspension containing CeO_2_ powder (particle size (d) = 0.5 μm), followed by ultrasonic cleaning and drying for subsequent microstructure observation and electrochemical testing.

### 2.2. Electrochemical Measurements

The electrochemical tests included the open circuit potential (OCP) measure ment, potentiodynamic polarization, and electrochemical impedance spectroscopy. All the electrochemical experiments were conducted on a CH Instrument 600C electrochemical station with a conventional three-electrode cell in 0.1 M H_2_SO_4_ solution at 25 °C. A platinum sheet and saturated calomel electrode were used as the counter and reference electrodes, respectively. On the working electrodes, an electrochemically active surface area of 1 cm^2^ was maintained. Before performing the electrochemical impedance spectroscopy (EIS) tests, the specimens were immersed in solution until a stable OCP was obtained. EIS was performed in a frequency range from 0.01 Hz to 100 kHz with amplitude of 5 mV. The potentiodynamic polarization curves of the specimens were obtained in 0.1 M H_2_SO_4_ with a sweep scanning rate of 1 mV/s.

### 2.3. Sample Characterisation

Phase identification was conducted with a D/max2500pc X-ray diffractometer with Cu-*K*α radiation (*λ* = 0.154 nm). The metallographic samples were cut, grounded, and polished according to the standard procedures and then etched with a solution consisting of H_2_O_2_ (10 mL), HCl (16 mL), and methyl alcohol (24 mL) for 3–5 s. The morphologies of the sample surfaces were observed by an optical microscope (OM) (Leica DM2700P). Microstructural observations were made using a scanning electron microscope (SEM) (Nova Nano SEM 230, FEI, Hillsboro, OR, USA) equipped with an energy-dispersive spectrometer (EDS). Electron backscatter diffraction (EBSD) measurements were performed using SEM (Helios Nanolab 600i, FEI, Hillsboro, OR, USA).

## 3. Results

### 3.1. Characterisation of the Initial Microstructure

Figure 1 shows the initial microstructures and a schematic diagram of the samples, with Figure 1a,b respectively showing OM images of the XY- and YZ-planes of the sample. The laser scanning track formed during the LMD process can be seen in Figure 1a. In the scale structure shown in Figure 1b, the contour of the melt pool is clear, which is attributed to the layer-by-layer deposition manner of the LMD process. Metallurgical defects are mainly distributed at the edge of the scanning track and the overlap regions of the two adjacent melt pools (See in Figure 1a,b). Considering their three-dimensional spatial relationship, these two locations are essentially coincident (Figure 1f).

SEM images of the areas in Figure 1a,b are shown in Figure 1c,d, respectively. The dendritic sub-grains are mainly distributed at the edge of the scanning track, whereas cellular sub-grains are primarily distributed inside the scanning track (see in Figure 1c). The cellular sub-grains are mainly distributed in the center of the molten pool, whereas dendritic sub-grains are mainly distributed at the bottom and edge of the melt pool (see in Figure 1d). Figure 1e shows an SEM image of the area in the center of the melt pool. The EDS maps of the different elements are shown in Figure 1e_1_–e_4_: All four main elements are evenly distributed in this region, and there is no element segregation at the grain boundaries. Generally, the segregation of elements would negatively impact the corrosion resistant properties. Due to the characteristics of high heating/cooling rate of laser processing technology, the products processed by this technology usually have fine microstructure and uniform element distribution.

**Figure 2:**
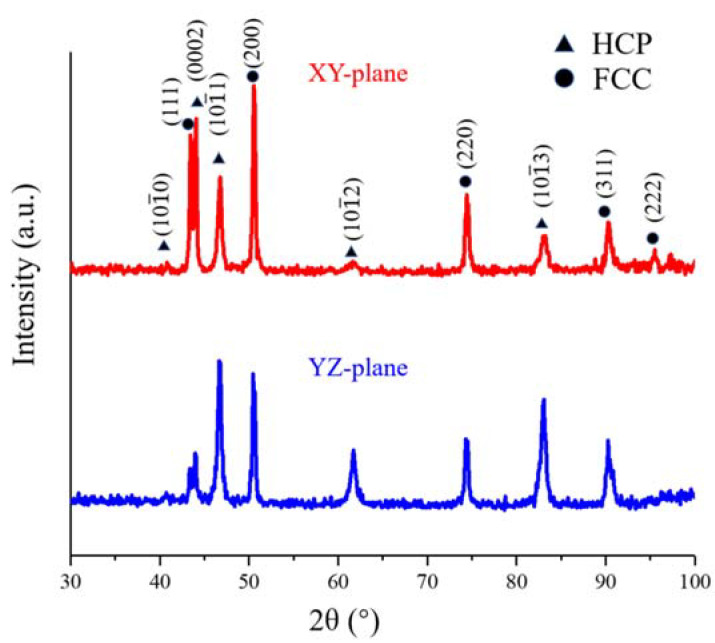
XRD patterns of the different planes of the LMD-produced Fe_50_Mn_30_Co_10_Cr_10_ HEA.

Figure 2 shows the X-ray diffraction (XRD) patterns at XY- and YZ-planes of this HEA, and both hexagonal close-packing (HCP) and FCC phases are recognized.

**Figure 3:**
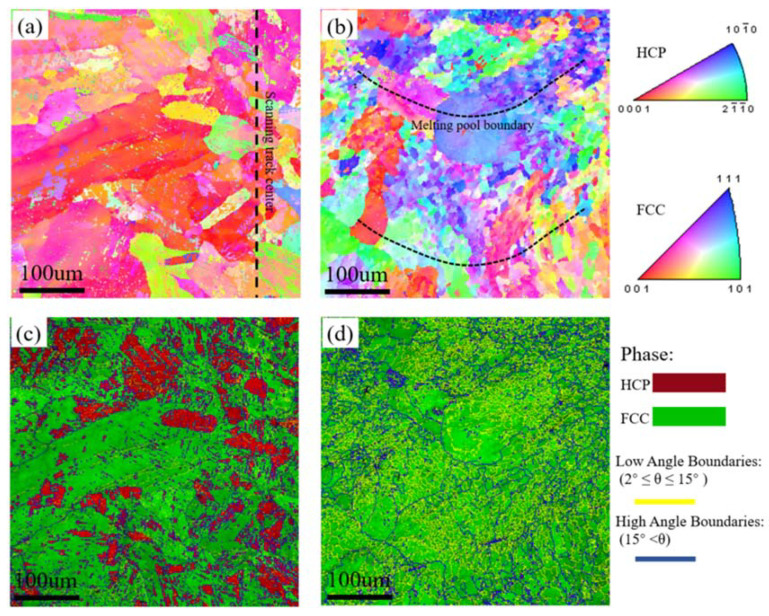
EBSD inverse pole figures (IPFs) and IQ + phase maps of the LMD sample: (**a**,**c**) XY-plane and (**b**,**d**) YZ-plane.

EBSD measurements were performed for further characterizing the microstructures of LMD samples, and the results are presented at Figure 3. Figure 3a,b show the inverse pole figure (IPF) maps, and Figure 3c,d show the IQ + phase maps. In Figure 3a,b, no obvious texture is evident on the XY- or YZ-plane of the sample. Grain size varied at different regions of the scanning track as observed in Figure 3a, fine equiaxed grains locate in the center of the scanning track and coarse columnar grains distribute at the edges. There is also a significant difference in the grain sizes at melt pool. In Figure 3b, we can see that the grains distributed at the boundary of the melt pool are significantly coarser than those at the center. During LMD processing, the cooling rate is different at different areas of the sample which directly controls the grain size. At the center of the molten pool, a higher temperature and cooling rate are generally expected, which thus leads to a high nucleation rate, grain growth is subsequently hindered along with grain refinement. Due to the lower temperature and slower cooling rate at the edge of the melt pool, the grains can grow epitaxially along the temperature gradient (toward the center of the melt pool), resulting in larger grains.

The phase distributions on the XY- and YZ-planes of the sample (see in Figure 3c,d), where the FCC phase is the dominant component at both the XY- and YZ-planes, while the HCP phase is mainly concentrated on the XY-plane.

**Figure 4:**
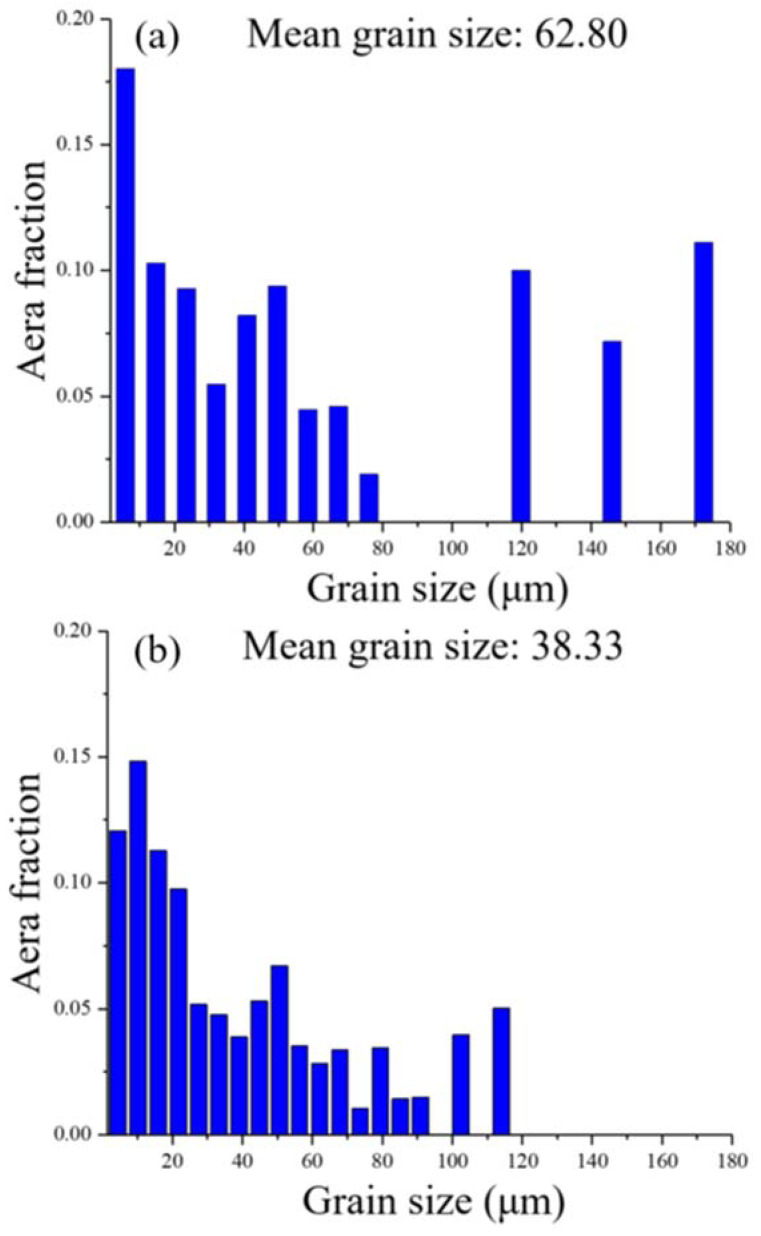
Grain size distributions of the LMD sample: (**a**) XY-plane and (**b**) YZ-plane.

Figure 4 shows the grain size distributions on the XY- and YZ-planes of the sample. The proportion of coarse grains (diameter greater than 100 μm) distributed on the XY-plane is significantly greater than that on the YZ-plane, and grains larger than 120 μm could only be identified at the XY-plane. In addition, the mean size of the grains distributed on the XY-plane is larger than that on the YZ-plane.

**Figure 5:**
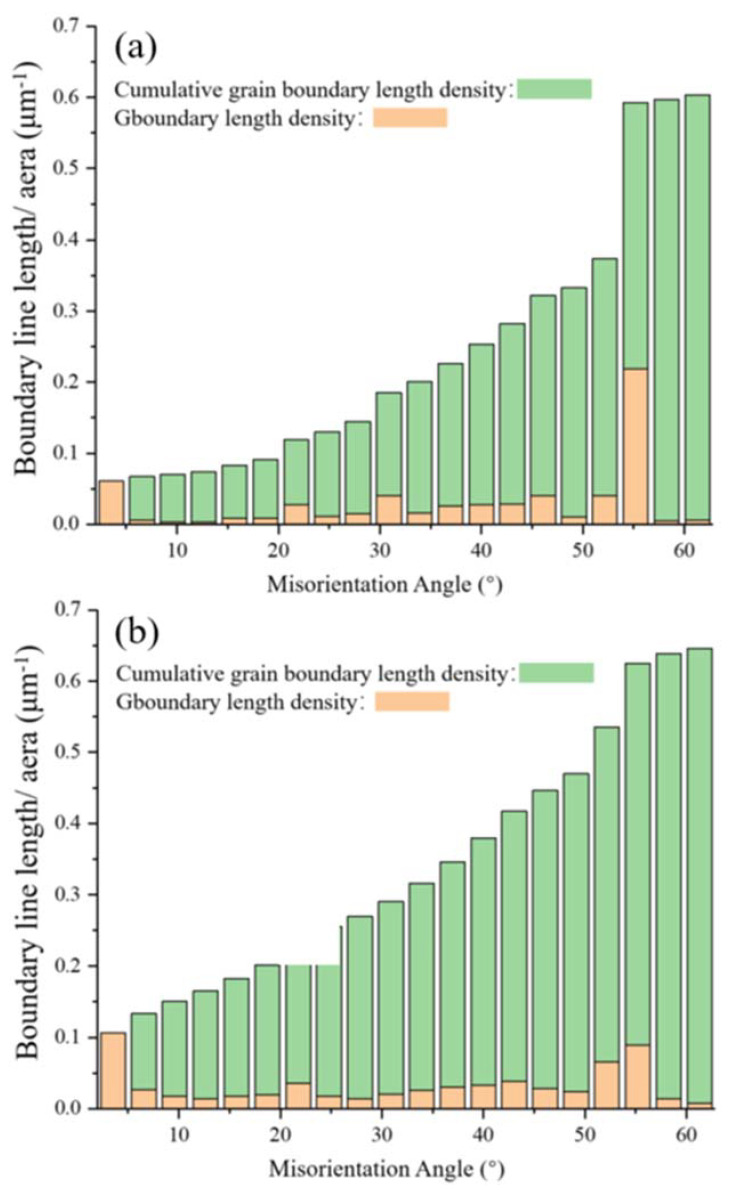
Distributions of grain boundary length density and cumulative grain boundary length density as a function of misorientation angle on the (**a**) XY-plane and (**b**) YZ-plane of the LMD sample.

Figure 5a,b show the grain-boundary-length densities and cumulative grain-boundary-length densities of the XY- and YZ-plane samples in the corresponding misorientation angle range, respectively. The grain boundaries with both low misorientation angles (0–10°) and high misorientation angles (50–60°) have greater density in the two planes. Among them, the grain-boundary density in the range of the high misorientation angle (50–60°) on the YZ-plane is highest, which is related to the higher proportion of large grains on the YZ-plane. These results show that the grain-boundary density on the YZ-plane is higher than that on the XY-plane and is inversely proportional to the mean grain size. Since the XY-plane has a higher proportion of large grains and a lower grain-boundary density, its area of high grain-boundary density will be significantly smaller than that of the YZ-plane.

### 3.2. Electrochemical Analyses

#### 3.2.1. Open Circuit Potential Measurement

**Figure 6:**
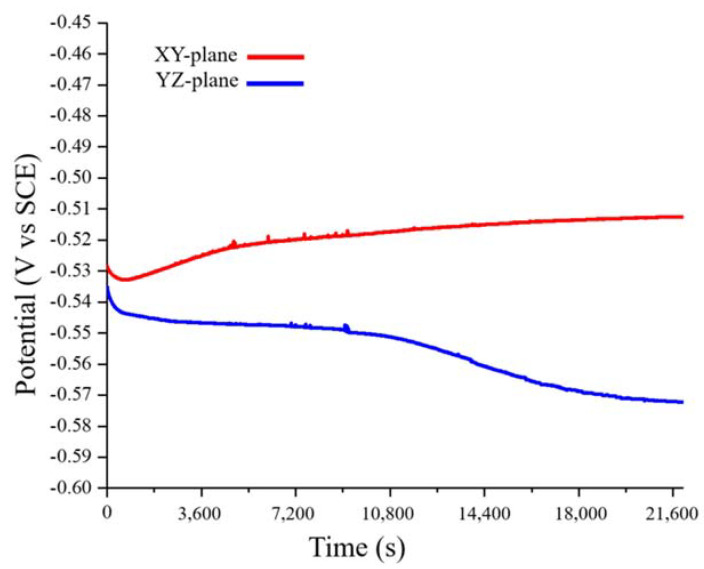
Open circuit potential (vs. SCE) of the LMD HEA in 0.1 M H_2_SO_4_ at 25 °C.

Figure 6 shows the variation with time of the OCPs of the XY- and YZ-plane samples. The OCP of the XY-plane sample made a short negative shift only at the beginning of the test, whereas that of the YZ-plane sample made a negative shift throughout the entire test. This may be related to the region with a high grain-boundary density in the sample. In general, high grain-boundary density will increase the anodic reaction, resulting in a negative OCP shift [34]. From Figure 3, Figure 4 and Figure 5, we can conclude that the YZ-plane sample has a larger area than the XY- plane with a high grain-boundary density (Figure 3). This is consistent with the long-term decrease in the OCP of the YZ-plane. Lastly, the OCPs of the XY- and YZ-planes are stable at approximately −0.51 V and −0.57 V, respectively.

#### 3.2.2. Potentiodynamic Polarization

**Figure 7:**
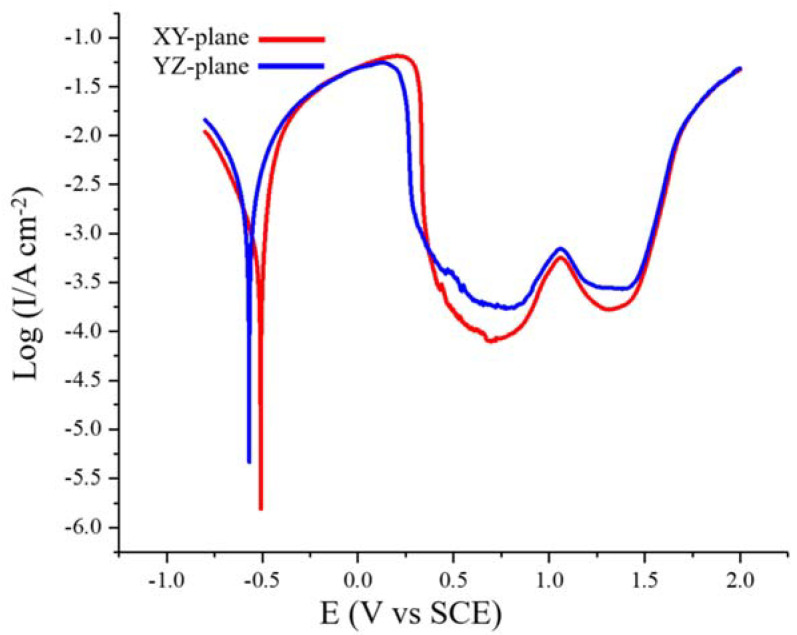
Potentiodynamic polarization curves of the SLM-produced Fe_50_Mn_30_Co_10_Cr_10_ HEA in 0.1 M H_2_SO_4_ solution at 25 °C.

Figure 7 shows the potentiodynamic polarization curves of the XY- and YZ-plane samples in the 0.1 M H_2_SO_4_ solution at a scan rate of 1 mV/s. The corrosion potential, *E*_corr_, the corrosion current density, *I*_corr_, the cathode and anode Tafel slopes, *β*_c_ and *β*_a_, and the polarization resistance, *R*_p_, were determined by Tafel fitting. The critical anodic current density required for passivation, *I*_crit_, the primary passivation potential, *E*_pp_, the repassivation potential, *E*_rp_, and the breakdown potentials, *E*_b_, were obtained from the potentiodynamic polarization curves. All the parameters are shown in Table 2. We can see that the XY-plane has higher *E*_corr_ and *R*_p_ values and a lower *I*_corr_ value. Generally, higher *E*_corr_ and *R*_p_ values and smaller *I*_corr_ values indicate better corrosion resistance [35,36], which means that the XY-plane sample has better corrosion resistance. However, The YZ-plane has lower *E*_pp_ and *I*_crit_ than the XZ-plane, which indicates that the YZ-plane is more likely to be passivated. In addition, the *E*_rp_ and *E*_b_ of the XY- and YZ-plane are equivalent.

#### 3.2.3. Electrochemical Impedance Spectroscopy

**Figure 8:**
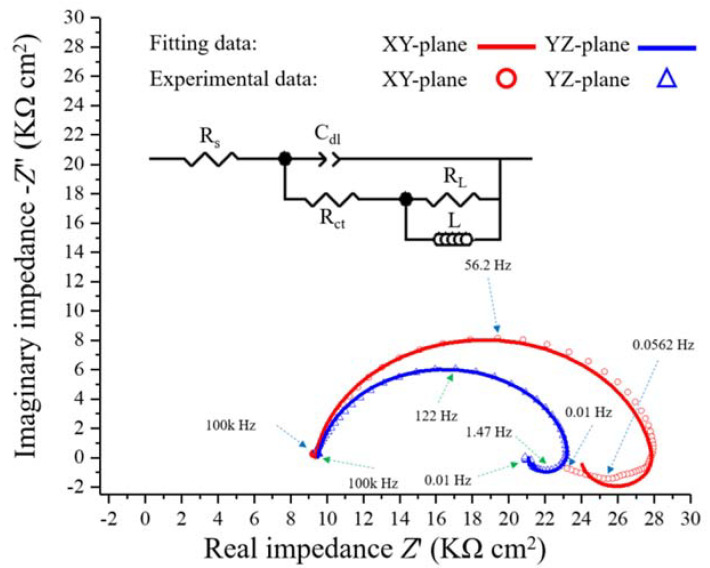
Complex plane impedance plots for the LMD-produced Fe_50_Mn_30_Co_10_Cr_10_ HEA in 0.1 M H_2_SO_4_ solution at 25 °C.

Figure 8 shows the complex plane impedance curves of the XY- and YZ-plane samples in the 0.1 M H_2_SO_4_ solution under OCP conditions at 25 °C. A depressed charge transfer semicircle at high frequency (HF) and a clear induction loop at low frequency (LF) can be observed. The semicircle at HF is attributed to the time constant of the double-layer capacitance and the charge transfer [37,38,39]. With the larger radius of the semicircle in the HF region, the charge transfer resistance is greater together with a better corrosion resistance. The inductive behavior in the LF region is attributed to the relaxation process associated with the adsorbed protons and sulfate ions [40,41]. Similar impedance curves were obtained in Ye et al. for the corrosion performance of the CrMnFeCoNi HEA [42]. The same equivalent circuit (EC) model was used to fit all the EIS parameters, which is shown in the inset in Figure 8.

In this EC model, *CPE* represents the constant phase element of electric double-layer capacitance, including the exponential quantity, *n*, and exponential coefficient, *Q*. *R*_s_ represents the electrolyte resistance, *R*_ct_ is the charge transfer resistance, *R*_L_ is the resistance related to the induction process and *L* is the pseudo-inductance of this process [43]. Table 3 lists all the parameter values obtained by the EC model. As shown in the table, the *R*_ct_ value of the XY-plane sample is larger than that of the YZ-plane sample, which indicates that the former has better corrosion resistance.

### 3.3. Morphology of the Corroded Surfaces

**Figure 9:**
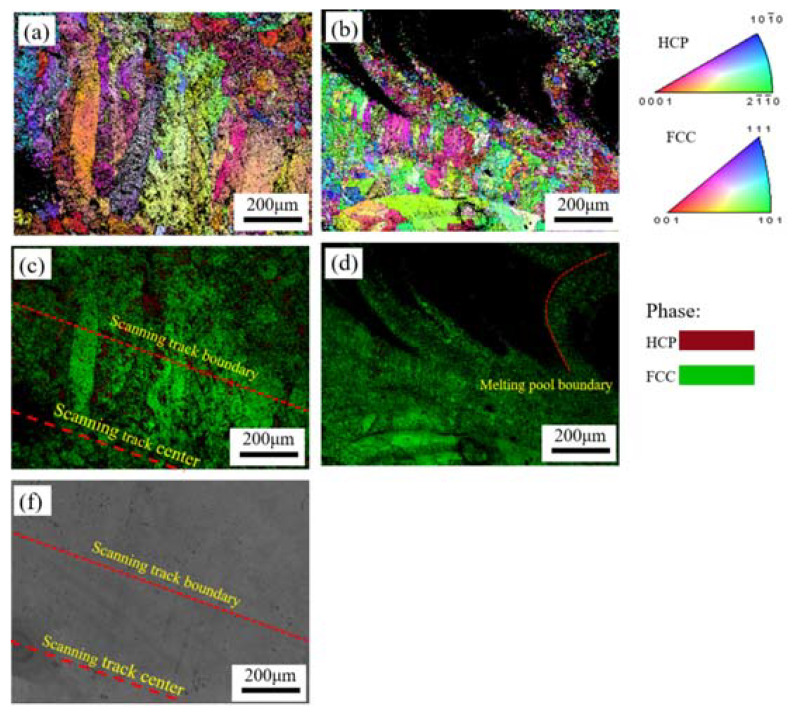
EBSD inverse pole figures (IPFs), IQ + phase maps and SEM image of the LMD HEA after electrochemical tests: (**a**,**c**,**f**) XY-plane and (**b**,**d**) YZ-plane.

Figure 9 shows EBSD images of the samples obtained after the electrochemical tests. Figure 9a,b show IPF maps of the XY- and YZ-planes, and Figure 9c,d show the respective IQ + phase maps. To more clearly show the distribution of different-size grains in the scanning track, Figure 9f shows an SEM image of the XY-plane in Figure 9a,c. Figure 9c,f reveal that coarse columnar grains are distributed at the scanning track edge, and equiaxed grains are distributed in the center of the scanning track. It is evident that the central area of the scanning track has experienced more severe general corrosion attacks than the edges. In Figure 9b,d, we can observe that common corrosion attacks are concentrated in the central area of the melt pool, and that the boundary of the melt pool has experienced less corrosion. Overall, general corrosion occurred in the center of the melt pool and scanning track, where fine grains are precisely distributed. In addition, the YZ-plane was subjected to more serious corrosion attack than the XY-plane, which is closely related to the fact that the YZ-plane has more fine grains than the XY-plane.

**Figure 10:**
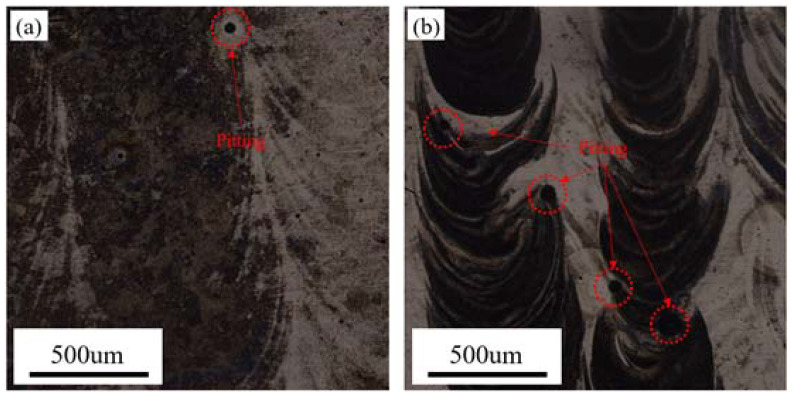
OM images of the LMD HEA after electrochemical tests: (**a**) XY-plane and (**b**) YZ-plane.

Figure 10 shows OM images of the samples after the electrochemical tests, in which there is obvious pitting. The pitting mainly distributed at the edge of the scanning track as well as boundary of the melt pool, respectively. Considering the three-dimensional spatial relationships of the sample, the edge of the scanning track is essentially overlapping the area of the molten pool boundary. This area coincides with the area in which metallurgical defects are concentrated, as shown in Figure 1, because metallurgical defects provide initial sites for pitting.

**Figure 11:**
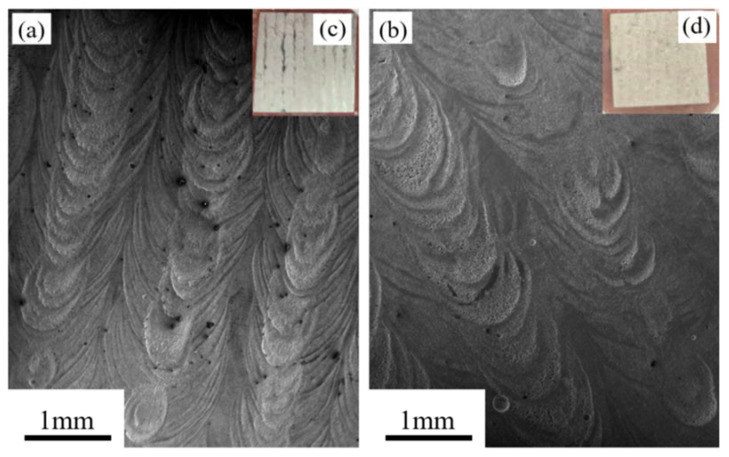
SEM images and macroscopic morphologies of the LMD HEA after electrochemical tests: (**a**,**c**) XY-plane and (**b**,**d**) YZ-plane.

Figure 11a,b show the respective SEM images of the XY- and YZ-plane samples soaked in 0.1 M H_2_SO_4_ for 12 h. Figure 11c,d show the respective macroscopic morphologies of Figure 11a,b. In Figure 11a,b, we can see that both planes have textures associated with uneven corrosion. The degree of corrosion changed at different regions on the two planes. Some regions are obviously sunk due to serious corrosion, while others are relatively flat due to mild corrosion. Compared with the XY- plane, the corrosion on the YZ-plane is more uniform. The metallic luster of the striped corrosion areas on the XY-plane can be observed in Figure 11c, which indicates that this area is less corroded. Although the texture of the YZ-plane due to uneven corrosion is also evident in Figure 11d, no metallic luster is evident. Overall, based on their surface morphologies after corrosion, we can conclude that the XY-plane has better corrosion resistance than the YZ-plane.

## 4. Discussion

The melting and solidification dynamics in LMD are very different from those in traditional manufacturing technologies [44,45]. Rapid melting and solidification followed by cyclic heating and cooling during subsequent layer depositions yields microstructures that differ from those of traditionally manufactured parts [46,47,48,49,50]. This difference leads to different corrosion properties, such as the uneven corrosion of the Fe_50_Mn_30_Co_10_Cr_10_ HEA fabricated by LMD.

In this study, we found the corrosion behavior of the LMD HEA to be closely related to its microstructure. General corrosion tended to occur in areas with a high grain-boundary density rich in fine grains, whereas pitting corrosion tended to occur at melt pool boundaries (MPBs). The characteristics of LMD processing lead to regularly distributed regions with high grain-boundary density and MPBs in the sample. This results in the regular distribution of corrosion areas on the sample, which ultimately produces striped corrosion morphology in Figure 11.

In the literature, there is no consensus regarding the influence of grain size on the corrosion resistance of ferrous alloys. Some authors argue that corrosion sensitivity decreases with decreases in grain size [51,52,53,54,55,56]. Others argue that decreases in grain size make ferrous alloys more vulnerable to corrosion [57,58,59]. Zeiger et al. stated that the relationship between grain size and corrosion depends on the environment. In active electrolytes, such as H_2_SO_4_ solution, the corrosion resistance of a fine-grain surface will decrease [60]. In this work, it was easy to observe differences in the corrosion resistance of regions with different grain sizes on the same plane. Whether on the XY-plane or YZ-plane, fine-grain areas were severely corroded. This finding is consistent with Zeiger et al.’s view.

In the samples prepared by LMD, the residual stress is concentrated at the MPBs. The presence of residual stress makes it easy for defects to occur in this area, which often serve as initial sites for pitting corrosion. In the 316L SS manufactured by AM, Zhou et al. also observed that pitting corrosion is most likely to occur at the MPB, which they attributed to the defects at the MPB [61]. Studies of Fe in acids have found that internal stress usually increases corrosion, due to either an increased cathodic reaction or anodic dissolution (more starting sites combined with higher surface activity) or the formation of defective oxides [51,52,62,63,64]. The combination of these factors makes MBPs prone to local corrosion.

In this study, grain size and residual stress are important factors affecting the corrosion properties of this HEA. The grain size can be controlled to a certain extent by adjusting the processing process, thus affecting the corrosion performance of the product. However, this may lead to a decline in the mechanical properties of the HEA. Therefore, reducing the residual stress of the HEA may be a suitable way to improve the corrosion performance of this HEA. Salvati et al. proposed a numerical modelling of the residual stresses and strains within mechanical components [65], which can be used to evaluate the residual strain in AM products. According to the evaluation, mechanical or thermal treatment can be used to reduce the local residual stress of the product, thereby reducing the occurrence of pitting corrosion.

## 5. Conclusions

In this study, the Fe_50_Mn_30_Co_10_Cr_10_ dual-phase HEA was successfully prepared by the LMD process. Electrochemical methods were used to study the corrosion behaviors of the different planes of this HEA in a 0.1 M H_2_SO_4_ solution. XRD, OM, SEM, and EBSD techniques were used to characterize the microstructures and surface morphologies of the different planes of this HEA before and after the electrochemical tests. The following conclusions can be drawn:

(1) Due to the characteristics of LMD processing, the microstructure of the product is regularly distributed over its surface, which results in the regular distribution of corrosion areas, and ultimately an uneven strip-like corrosion morphology.

(2) The grain size of this LMD HEA is the key factor determining the general corrosion. Areas with high grain-boundary density rich in small grains are vulnerable to general corrosion and have poor corrosion resistance.

(3) The XY-plane of this LMD HEA has better corrosion resistance than the YZ-plane, which is mainly attributable to the fact that the XY-plane has more coarse grains than the YZ-plane, and thus has a smaller area with a high grain-boundary density that is prone to corrosion.

(4) The pitting in this LMD HEA mainly occurred at the MBP, which is attributed to the concentration of residual stress and abundant defects that may provide the initial sites for pitting.

## Figures and Tables

**Table 1:** Detailed chemical compositions (at.%) of the Fe_50_Mn_30_Co_10_Cr_10_ powder.

Alloys	Fe	Mn	Co	Cr	Ni	Si	Zr	C	O	S	P
Fe_50_Mn_30_Co_10_Cr_10_	52.3	28.2	9.44	9.89	0.0008	<0.001	<0.00001	<0.0002	0.003	0.00019	0.00016

**Table 2:** Electrochemical parameters extracted and calculated from potentiodynamic polarization curves in 0.1 M H_2_SO_4_ solution.

Sample	*E*_corr_ (mV)	*I*_corr_ (mA cm^−2^)	*β*_a_ (mV dec^−1^)	−*β*_c_ (mV dec^−1^)	*R*_p_ (Ω cm^−2^)	*E*_pp_ (mV)	*I*_crit_ (mA cm^−2^)	*E*_rp_ (mV)	*E*_b_ (mV)
XY-plane	−509	0.6216	66.25	174.64	16.6	20	65.14	1060	1500
YZ-plane	−570	2.513	177.59	207.60	33.6	13	55.68	1060	1500

**Table 3:** Parameters of the EC model obtained from simulations based on the EIS experimental data.

Sample	*R*_s_ (Ω cm^−2^)	*Q*_dl_ (l0^−4^ Ω^−1^ cm^−2^ s^n^)	*n*_dl_	*R*_ct_ (Ω cm^−2^)	*R*_L_ (Ω cm^−2^)	*L* (H cm^−2^)	*χ*^2^ × 10^−3^
XY-plane	9.352	2.41	0.90778	14.63	3.952	7.23	3.035
YZ-plane	9.486	1.61	0.90407	11	2.947	0.2	2.787

## Data Availability

The raw/processed data required to reproduce these findings cannot be shared at this time as the data also forms part of an ongoing study.
